# Supplementary material for: Modeling glioblastoma heterogeneity as a dynamic network of cell states
Source: Mol Syst Biol. 2021 Sep 16;17(9):e10105. doi: 10.15252/msb.202010105 (PMC8444284; doi:10.15252/msb.202010105)
Supplement: Supplementary file 6 — Source Data for Figure 5 [file MSB-17-e10105-s004.zip › Figure5A_sourcedata/GSEA_3071/hallmarks_stateI.GseaPreranked.1623742531165/HALLMARK_EPITHELIAL_MESENCHYMAL_TRANSITION.html]

Details for gene set HALLMARK\_EPITHELIAL\_MESENCHYMAL\_TRANSITION[GSEA]

|  || Dataset | state13071 |
| Phenotype | NoPhenotypeAvailable |
| Upregulated in class | na\_pos |
| GeneSet | HALLMARK\_EPITHELIAL\_MESENCHYMAL\_TRANSITION |
| Enrichment Score (ES) | 0.5769789 |
| Normalized Enrichment Score (NES) | 3.5389493 |
| Nominal p-value | 0.0 |
| FDR q-value | 0.0 |
| FWER p-Value | 0.0 |
Table: GSEA Results Summary

  

Fig 1: Enrichment plot: HALLMARK\_EPITHELIAL\_MESENCHYMAL\_TRANSITION      
 Profile of the Running ES Score & Positions of GeneSet Members on the Rank Ordered List

  

| PROBE | GENE SYMBOL | GENE\_TITLE | RANK IN GENE LIST | RANK METRIC SCORE | RUNNING ES | CORE ENRICHMENT || 1 | SPP1 |  |  | 0 | 2.808 | 0.0268 | Yes |
| 2 | MGP |  |  | 1 | 2.652 | 0.0521 | Yes |
| 3 | COL3A1 |  |  | 3 | 2.646 | 0.0771 | Yes |
| 4 | LUM |  |  | 4 | 2.203 | 0.0981 | Yes |
| 5 | DCN |  |  | 5 | 2.010 | 0.1173 | Yes |
| 6 | COL1A1 |  |  | 6 | 2.008 | 0.1365 | Yes |
| 7 | PDGFRB |  |  | 9 | 1.893 | 0.1540 | Yes |
| 8 | FN1 |  |  | 11 | 1.769 | 0.1705 | Yes |
| 9 | COL6A3 |  |  | 13 | 1.717 | 0.1866 | Yes |
| 10 | COL5A1 |  |  | 14 | 1.706 | 0.2029 | Yes |
| 11 | PCOLCE |  |  | 15 | 1.696 | 0.2191 | Yes |
| 12 | TGFBI |  |  | 17 | 1.674 | 0.2348 | Yes |
| 13 | BGN |  |  | 24 | 1.568 | 0.2480 | Yes |
| 14 | MMP14 |  |  | 27 | 1.526 | 0.2620 | Yes |
| 15 | COL4A1 |  |  | 28 | 1.524 | 0.2765 | Yes |
| 16 | POSTN |  |  | 32 | 1.494 | 0.2899 | Yes |
| 17 | MATN2 |  |  | 34 | 1.483 | 0.3038 | Yes |
| 18 | COL11A1 |  |  | 37 | 1.468 | 0.3172 | Yes |
| 19 | TIMP1 |  |  | 47 | 1.381 | 0.3277 | Yes |
| 20 | COL5A2 |  |  | 48 | 1.373 | 0.3408 | Yes |
| 21 | CTHRC1 |  |  | 49 | 1.370 | 0.3539 | Yes |
| 22 | CDH11 |  |  | 59 | 1.296 | 0.3636 | Yes |
| 23 | EDIL3 |  |  | 62 | 1.251 | 0.3750 | Yes |
| 24 | COL6A2 |  |  | 67 | 1.237 | 0.3856 | Yes |
| 25 | COL4A2 |  |  | 73 | 1.203 | 0.3956 | Yes |
| 26 | LRP1 |  |  | 84 | 1.160 | 0.4038 | Yes |
| 27 | NNMT |  |  | 88 | 1.132 | 0.4137 | Yes |
| 28 | COL12A1 |  |  | 91 | 1.117 | 0.4238 | Yes |
| 29 | THY1 |  |  | 94 | 1.114 | 0.4338 | Yes |
| 30 | SPARC |  |  | 106 | 1.067 | 0.4408 | Yes |
| 31 | ITGAV |  |  | 117 | 1.029 | 0.4476 | Yes |
| 32 | FBN1 |  |  | 138 | 0.944 | 0.4508 | Yes |
| 33 | INHBA |  |  | 140 | 0.941 | 0.4594 | Yes |
| 34 | SNAI2 |  |  | 142 | 0.940 | 0.4681 | Yes |
| 35 | SERPINH1 |  |  | 157 | 0.902 | 0.4726 | Yes |
| 36 | TGFB1 |  |  | 165 | 0.884 | 0.4790 | Yes |
| 37 | P3H1 |  |  | 186 | 0.849 | 0.4812 | Yes |
| 38 | COL8A2 |  |  | 192 | 0.842 | 0.4878 | Yes |
| 39 | ITGA2 |  |  | 194 | 0.839 | 0.4955 | Yes |
| 40 | VCAN |  |  | 197 | 0.833 | 0.5029 | Yes |
| 41 | CALD1 |  |  | 201 | 0.824 | 0.5099 | Yes |
| 42 | COL7A1 |  |  | 223 | 0.779 | 0.5111 | Yes |
| 43 | ECM2 |  |  | 257 | 0.727 | 0.5083 | Yes |
| 44 | COL5A3 |  |  | 258 | 0.727 | 0.5153 | Yes |
| 45 | EMP3 |  |  | 260 | 0.726 | 0.5219 | Yes |
| 46 | IGFBP4 |  |  | 262 | 0.725 | 0.5285 | Yes |
| 47 | PTX3 |  |  | 267 | 0.716 | 0.5342 | Yes |
| 48 | PLOD1 |  |  | 271 | 0.713 | 0.5401 | Yes |
| 49 | DAB2 |  |  | 277 | 0.702 | 0.5453 | Yes |
| 50 | MMP2 |  |  | 284 | 0.697 | 0.5502 | Yes |
| 51 | TPM2 |  |  | 320 | 0.655 | 0.5462 | Yes |
| 52 | BMP1 |  |  | 346 | 0.631 | 0.5448 | Yes |
| 53 | SERPINE2 |  |  | 364 | 0.624 | 0.5458 | Yes |
| 54 | GPX7 |  |  | 368 | 0.617 | 0.5508 | Yes |
| 55 | ITGB1 |  |  | 371 | 0.615 | 0.5561 | Yes |
| 56 | FLNA |  |  | 373 | 0.614 | 0.5616 | Yes |
| 57 | MATN3 |  |  | 379 | 0.609 | 0.5660 | Yes |
| 58 | MYL9 |  |  | 417 | 0.576 | 0.5606 | Yes |
| 59 | TPM1 |  |  | 419 | 0.575 | 0.5658 | Yes |
| 60 | NTM |  |  | 427 | 0.572 | 0.5692 | Yes |
| 61 | PPIB |  |  | 442 | 0.555 | 0.5704 | Yes |
| 62 | NOTCH2 |  |  | 446 | 0.551 | 0.5747 | Yes |
| 63 | BASP1 |  |  | 457 | 0.544 | 0.5770 | Yes |
| 64 | FSTL1 |  |  | 505 | 0.504 | 0.5679 | No |
| 65 | LAMA2 |  |  | 506 | 0.503 | 0.5728 | No |
| 66 | HTRA1 |  |  | 555 | 0.477 | 0.5632 | No |
| 67 | LOXL2 |  |  | 589 | 0.459 | 0.5578 | No |
| 68 | VCAM1 |  |  | 603 | 0.450 | 0.5583 | No |
| 69 | RHOB |  |  | 609 | 0.447 | 0.5611 | No |
| 70 | THBS1 |  |  | 613 | 0.446 | 0.5645 | No |
| 71 | IL15 |  |  | 635 | 0.439 | 0.5625 | No |
| 72 | LAMC1 |  |  | 640 | 0.439 | 0.5655 | No |
| 73 | FERMT2 |  |  | 704 | 0.410 | 0.5508 | No |
| 74 | GJA1 |  |  | 727 | 0.403 | 0.5482 | No |
| 75 | COPA |  |  | 771 | 0.392 | 0.5393 | No |
| 76 | TIMP3 |  |  | 773 | 0.391 | 0.5427 | No |
| 77 | ADAM12 |  |  | 776 | 0.390 | 0.5459 | No |
| 78 | DST |  |  | 804 | 0.380 | 0.5415 | No |
| 79 | LOXL1 |  |  | 857 | 0.363 | 0.5297 | No |
| 80 | ITGA5 |  |  | 874 | 0.357 | 0.5284 | No |
| 81 | GPC1 |  |  | 882 | 0.354 | 0.5297 | No |
| 82 | SLIT3 |  |  | 911 | 0.348 | 0.5248 | No |
| 83 | PMP22 |  |  | 1069 | 0.312 | 0.4816 | No |
| 84 | ITGB3 |  |  | 1102 | 0.306 | 0.4751 | No |
| 85 | VIM |  |  | 1105 | 0.305 | 0.4774 | No |
| 86 | MYLK |  |  | 1204 | 0.287 | 0.4513 | No |
| 87 | CAPG |  |  | 1222 | 0.285 | 0.4490 | No |
| 88 | PTHLH |  |  | 1279 | 0.272 | 0.4351 | No |
| 89 | PLOD2 |  |  | 1304 | 0.268 | 0.4306 | No |
| 90 | GLIPR1 |  |  | 1346 | 0.260 | 0.4210 | No |
| 91 | CD44 |  |  | 1430 | -0.251 | 0.3989 | No |
| 92 | TGM2 |  |  | 1444 | -0.252 | 0.3975 | No |
| 93 | VEGFA |  |  | 1644 | -0.273 | 0.3415 | No |
| 94 | ENO2 |  |  | 1707 | -0.281 | 0.3260 | No |
| 95 | APLP1 |  |  | 2066 | -0.328 | 0.2237 | No |
| 96 | MFAP5 |  |  | 2104 | -0.335 | 0.2160 | No |
| 97 | PMEPA1 |  |  | 2107 | -0.336 | 0.2186 | No |
| 98 | FBN2 |  |  | 2221 | -0.357 | 0.1887 | No |
| 99 | FZD8 |  |  | 2544 | -0.426 | 0.0980 | No |
| 100 | PDLIM4 |  |  | 2861 | -0.525 | 0.0099 | No |
| 101 | SFRP1 |  |  | 2926 | -0.559 | -0.0036 | No |
| 102 | DKK1 |  |  | 2992 | -0.596 | -0.0170 | No |
| 103 | GAS1 |  |  | 3009 | -0.607 | -0.0159 | No |
| 104 | SERPINE1 |  |  | 3050 | -0.634 | -0.0217 | No |
| 105 | SCG2 |  |  | 3130 | -0.703 | -0.0382 | No |
| 106 | PFN2 |  |  | 3252 | -0.852 | -0.0657 | No |
| 107 | MEST |  |  | 3307 | -0.940 | -0.0726 | No |
| 108 | FBLN1 |  |  | 3317 | -0.954 | -0.0662 | No |
| 109 | TNC |  |  | 3350 | -1.043 | -0.0656 | No |
| 110 | ID2 |  |  | 3374 | -1.110 | -0.0618 | No |
| 111 | DPYSL3 |  |  | 3377 | -1.119 | -0.0517 | No |
| 112 | MCM7 |  |  | 3384 | -1.147 | -0.0425 | No |
| 113 | IGFBP3 |  |  | 3433 | -1.384 | -0.0435 | No |
| 114 | CDH2 |  |  | 3439 | -1.439 | -0.0312 | No |
| 115 | CADM1 |  |  | 3485 | -1.943 | -0.0259 | No |
| 116 | IGFBP2 |  |  | 3509 | -3.484 | 0.0006 | No |
Table: GSEA details [plain text format]

  

Fig 2: HALLMARK\_EPITHELIAL\_MESENCHYMAL\_TRANSITION: Random ES distribution      
 Gene set null distribution of ES for **HALLMARK\_EPITHELIAL\_MESENCHYMAL\_TRANSITION**
